# Decoding astrocytic tryptophan metabolism in the pathogenesis of epilepsy: evidence from artificial intelligence-driven multi-omics and clinical validation

**DOI:** 10.3389/fnins.2026.1913179

**Published:** 2026-08-17

**Authors:** Wenhui Wang, Meizhen Sun

**Affiliations:** 1 Department of Neurology, Children’s Hospital of Shanxi, Shanxi Maternal and Child Health Hospital, The Affiliated Children’s Hospital of Shanxi Medical University, Taiyuan, Shanxi, China; 2 Department of Neurology, First Affiliated Hospital of Shanxi Medical University, Taiyuan, Shanxi, China

**Keywords:** artificial intelligence, astrocyte, drug repositioning, epilepsy, tryptophan metabolism

## Abstract

**Background:**

Epilepsy (EP) is a prevalent neurological disorder with complex etiology, often involving metabolic dysregulation. Emerging evidence highlights the role of tryptophan metabolism (TM) and astrocyte dysfunction in EP pathogenesis. This study aimed to decode the TM and astrocyte (TA)-related molecular signature and identify a central therapeutic target for EP.

**Methods:**

By integrating seven hippocampal bulk profiles (GSE28674, GSE256068, GSE57585, GSE163296, GSE63808, GSE90886, and GSE134697) from patients with EP using integrative bioinformatics pipelines, including limma, xCell, WGCNA, machine learning, and consensus clustering, we identified a TA-associated diagnostic signature and a molecular stratification model for EP. Next, the TA-associated hub gene was identified by SHAP analysis, and its molecular patterns in astrocytes were characterized using single-cell hippocampal data from patients with EP (GSE190452). In addition, the active-learning framework DrugReflector and molecular docking were used to identify a potential therapeutic agent targeting the TA-associated hub gene in the integrated GSE63808 and GSE90886 dataset. Finally, hippocampal tissue from patients with EP was used to examine expression of the TA-associated hub gene.

**Results:**

Four TA-associated shared DEGs were identified: CAT, TXNDC2, RBM27, and GPT. These four DEGs showed predictive value for EP. RBM27 was identified as the central pathogenic factor; it was upregulated and predominantly expressed in astrocytes. Drug prediction identified BRD-K04111260 as a promising compound targeting RBM27 for the treatment of EP.

**Conclusion:**

This study establishes a novel TA-associated molecular axis in EP pathogenesis, identifying RBM27 as a critical hub gene with strong diagnostic potential and therapeutic promise.

## 1 Introduction

Epilepsy (EP) is one of the most common serious neurological disorders, affecting approximately 50 million people worldwide (Thijs et al., 2019). It is characterized by an enduring predisposition to generate epileptic seizures, resulting from excessive, synchronous neuronal firing in the brain (Manford, 2017). The pathophysiological hallmarks include disrupted ion channel function, altered neurotransmitter balance, particularly an imbalance between glutamatergic excitation and GABAergic inhibition and structural abnormalities such as hippocampal sclerosis (Li C. et al., 2025). Despite the availability of numerous antiseizure medications, approximately one-third of patients continue to experience seizures, highlighting the urgent need for novel therapeutic targets based on a deeper understanding of underlying molecular mechanisms (Beghi et al., 2015).

Tryptophan metabolism (TM) is a critical biochemical pathway that generates several bioactive metabolites, including kynurenine, quinolinic acid, and serotonin (Correia and Vale, 2022). The kynurenine pathway, which accounts for over 90% of tryptophan catabolism, produces neuroactive intermediates that can modulate glutamatergic neurotransmission and neuroinflammation (Zhao et al., 2025a). In the context of EP, dysregulated TM has been increasingly implicated; elevated levels of neurotoxic quinolinic acid and altered kynurenic acid-to-quinolinic acid ratios have been reported in the cerebrospinal fluid and brain tissue of patients with EP (Chojnowski et al., 2025). Concurrently, astrocytes, the most abundant glial cells in the brain, play a pivotal role in maintaining synaptic homeostasis, providing metabolic support to neurons, and regulating neuroinflammation (Vezzani et al., 2022). Astrocyte dysfunction, including impaired glutamate uptake and aberrant release of inflammatory mediators, is a well-established contributor to seizure generation and propagation (Binder and Steinhäuser, 2021). Notably, astrocytes are the primary site of TM in the central nervous system, and dysregulation of astrocytic TM is emerging as a key driver of epileptogenesis.

This study was designed to bridge these observations. We hypothesized that a shared molecular signature linking TM and astrocyte (TA) underpins EP pathogenesis. To test this, we integrated bulk transcriptomic data from multiple EP cohorts, employed an ensemble machine-learning framework to construct a diagnostic model, and identified a central hub gene. Single-cell analysis provided cellular-resolution insight, and drug prediction coupled with clinical q-RT-PCR validation offered translational relevance.

## 2 Materials and methods

### 2.1 Bulk profile identification

Seven bulk transcriptome datasets derived from human hippocampal tissue of EP patients were retrieved from the GEO database, including GSE28674, GSE256068, GSE57585, GSE163296, GSE63808, GSE90886, and GSE134697 (Bando et al., 2021; He et al., 2024; Jiang et al., 2025; Li S. et al., 2025; Moreira-Filho et al., 2015; Naqvi et al., 2025; Wu et al., 2022). We integrated GSE28674, GSE57585 and GSE256068 for identification of 32 EP samples and 13 healthy controls (HCs). We integrated GSE63808 and GSE90886 for identification of 129 EP samples and 6 HCs. All datasets were integrated by sva package in R to correct for batch effects (Leek et al., 2012). GSE163296 included 22 EP samples. GSE134697 included 17 EP samples and 2 HCs. All datasets were normalized using the limma package in R (Ritchie et al., 2015). TM-associated gene list was identified in GeneCards database with relevance threshold greater than 1.

### 2.2 Limma analysis

In the integrated GSE28674, GSE57585, and GSE256068 dataset, differential expression analysis between EP and HC samples was performed using the limma package in R (Ritchie et al., 2015). Genes with an adjusted *p*-value < 0.05 and | log2 (Fold Change)| > 1 were designated as DEGs (Ritchie et al., 2015). The intersection of DEGs with TM-related genes yielded TM-associated DEGs. Their expression pattern was visualized in a heatmap using the pheatmap package in R (Bai et al., 2024). Protein-protein interaction (PPI) network analysis was performed using the STRING database (Szklarczyk et al., 2023). Functional enrichment analysis was conducted using the clusterProfiler package in R, referencing the KEGG and GO gene sets from the MSigDB database (Yu et al., 2012).

### 2.3 xCell and WGCNA analysis

The xCell package in R was used to infer the relative abundance of 64 cell types, including astrocytes, in the integrated GSE28674, GSE57585 and GSE256068 dataset (Angel et al., 2025). WGCNA was performed using the WGCNA package in R to identify modules correlated with the EP phenotype and astrocyte score (Langfelder and Horvath, 2008). A soft-thresholding power (β) was selected to achieve a scale-free topology fit index *R*^2^ > 0.85 (Langfelder and Horvath, 2008). The adjacency matrix was transformed into a TOM, and modules were identified via hierarchical clustering (minimum module size = 60) (Langfelder and Horvath, 2008). Pearson correlation between module eigengenes and both EP status and astrocyte score was calculated (Langfelder and Horvath, 2008). The module with the highest positive correlation with both traits was selected as the key module (Langfelder and Horvath, 2008). Genes from this module were intersected with the TM-associated DEGs to define the TA-associated shared DEGs.

### 2.4 Consensus clustering analysis

To identify molecular subtypes, consensus clustering of patients with EP in the GSE163296 dataset was performed based on expression of the TA-associated shared DEGs using the ConsensusClusterPlus package in R (Hu et al., 2022). The optimal number of clusters (k) was determined by evaluating the consensus cumulative distribution function (CDF) curves, delta area plots, and tracking plots (Hu et al., 2022). Differences in gene expression (performed by ComplexHeatmap in R), pathway enrichment (performed by GSEA using the clusterProfiler package referencing the KEGG and GO gene sets from the MSigDB database), and immune cell infiltration (performed by CIBERSORT using the immunedeconv package in R) between the identified subgroups were assessed (Chen et al., 2018; Gu, 2022; Yu et al., 2012).

### 2.5 Machine learning for diagnostic model construction

An ensemble machine-learning framework comprising 132 distinct classifier combinations was developed using the integrated GSE63808 and GSE90886 set (Wu et al., 2024). Each model was evaluated using 10-fold cross-validation repeated 3 times, with the AUC-index as the primary metric (Wu et al., 2024). The model with the highest mean AUC-index was selected as the optimal model (Wu et al., 2024). Its performance was externally validated using the GSE134697 dataset. ROC and PR curves were generated using the pROC package in R for the integrated GSE63808 and GSE90886 dataset and for GSE134697 (Robin et al., 2011). A nomogram and calibration curve were plotted using the rms package in R for the integrated GSE63808 and GSE90886 dataset. To interpret the model output, the SHAP algorithm was applied using the shapviz package in R to identify the top-contributing gene as TA-associated hub gene (Huang et al., 2025). Single-gene GSEA for the hub gene was performed in the integrated GSE63808 and GSE90886 set using the clusterProfiler package referencing the KEGG and GO gene sets from the MSigDB database (Yu et al., 2012).

### 2.6 Single-cell analysis

The single-cell RNA-seq dataset GSE190452, derived from hippocampal tissue of 4 EP patients, was processed using the Seurat package in R (Tan et al., 2023; Zhao et al., 2025b). Quality control (QC) involved filtering cells with feature counts between 200 and 5,000 and a mitochondrial gene percentage below 10% (Tan et al., 2023). Data were normalized using the SCTransform method (Tan et al., 2023). PCA was performed for dimensionality reduction, followed by graph-based clustering and UMAP and t-SNE visualization (Tan et al., 2023). Cell types were annotated using the SingleR package in R with the Human Primary Cell Atlas reference (Tan et al., 2023). Cell-cell communication analysis, including identification of ligand-receptor pairs, was performed using CellChat package in R (Fang et al., 2022). Activity of the TM pathway was assessed using the AUCell package in R, scoring each cell based on the curated TM gene set (Deng et al., 2021). Metabolic heterogeneity was evaluated using the scMetabolism package in R (Qing et al., 2024). Expression of the hub gene across cell types was visualized on a dot plot. An *in silico* knockout of the hub gene specifically in astrocytes was simulated using the scTenifoldKnk package in R (Jie et al., 2026). The top 10 perturbed genes were subjected to GeneMANIA analysis with GO and KEGG enrichment using clusterProfiler package in R referencing the KEGG and GO gene sets from the MSigDB database (Yu et al., 2012; Zhao et al., 2021). Differentiation trajectories of astrocytes were inferred using the Monocle2 package in R, with hub gene expression mapped along the pseudotime trajectory (Huang et al., 2024).

### 2.7 Drug screening and molecular docking

To identify potential therapeutic agents targeting the TA-associated molecular signature, the DrugReflector platform in R was employed (Ma, 2026). Using the differentially expressed genes between EP and HC in the integrated GSE63808 and GSE90886 training set as input, the platform integrated this signature with the cMAP drug perturbation database (Ma, 2026). Each compound was assigned a Drug-Target Association Score (DTAS) based on the connectivity of its expression signature to the query signature (Ma, 2026). The compound with the highest DTAS was selected (Ma, 2026). Its binding affinity with the hub gene protein product was then assessed via molecular docking using AutoDock Vina software (Zhou et al., 2026). The three-dimensional structure of the hub protein was retrieved from the PDB Database, and the compound structure was downloaded from PubChem database (Zhou et al., 2026). Binding energies were calculated, and the lowest energy conformation was visualized using PyMOL software (Zhou et al., 2026).

### 2.8 q-RT-PCR for clinical validation

To validate hub gene expression at the mRNA level, hippocampal tissue samples were collected from 6 EP patients and 6 age-matched controls, following ethical approval and informed consent. Total RNA was extracted using TRIzol reagent (Invitrogen, United States). One microgram of RNA was reverse-transcribed using a PrimeScript RT kit (Takara, Japan). Quantitative real-time PCR was performed using TB Green Premix Ex Taq II (Takara, Japan) on a LightCycler 480 II system (Roche, United States). Relative expression was calculated using the 2^−ΔΔ^Ct method with GAPDH as the endogenous control. The primers used in this study were as follows (5′–3′): RBM27 forward, 5′-GCTGAAGACCACCTCAACGA-3′; RBM27 reverse, 5′-TGGTCATGTAATCGGCTTGC-3′; GAPDH forward, 5′-GGAGCGAGATCCCTCCAAAAT-3′; GAPDH reverse, 5′-GGCTGTTGTCATACTTCTCATGG-3′.

### 2.9 Statistical analysis

All statistical analyses were performed using R version 4.2.2 or GraphPad Prism version 9.1. Comparisons between two groups were conducted using an unpaired Student’s *t*-test for normally distributed data or a Mann-Whitney U test for non-normally distributed data. A *p* < 0.05 was considered statistically significant.

## 3 Results

### 3.1 Identification of TM-associated DEGs for EP patients

**FIGURE 1:**
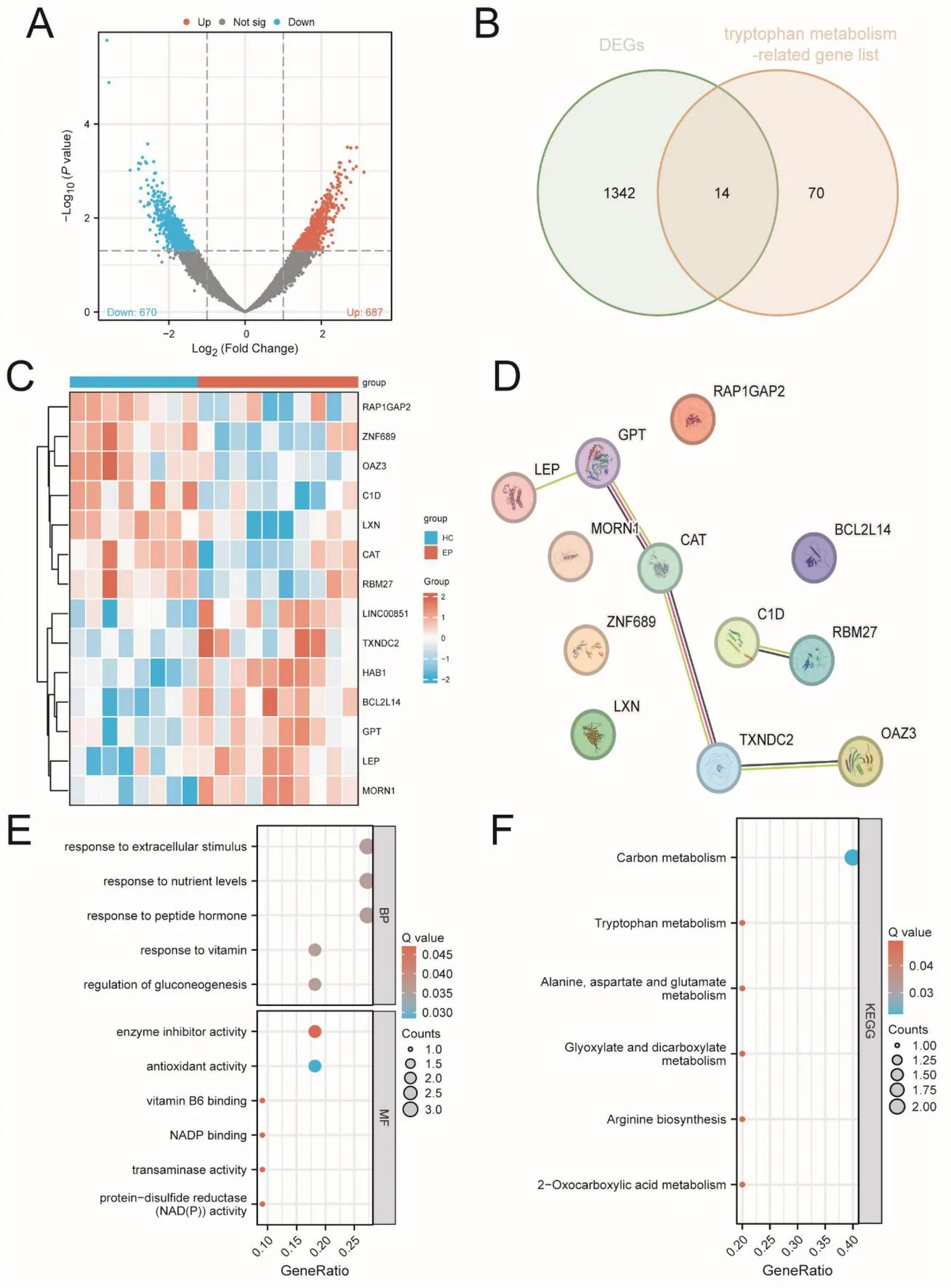
Identification of TM-associated DEGs in EP. **(A)** Volcano plot of DEGs in integrated GSE28674, GSE57585, and GSE256068 profiles. **(B)** Venn diagram showing the intersection of DEGs with the TM gene list, yielding 14 TM-associated DEGs. **(C)** Heatmap of TM-associated DEGs, showing distinct expression patterns between EP and HC. **(D)** STRING protein–protein interaction network of the TM-associated DEGs. **(E,F)** Dot plots of enriched GO and KEGG terms. Results with *p* < 0.05 or FDR < 0.05 were considered significant.

After preprocessing the integrated GSE28674, GSE57585, and GSE256068 profiles, differential expression analysis identified 687 upregulated and 670 downregulated DEGs between EP and HC samples (Figure 1A). A targeted intersection with the TM gene set yielded 14 TM-associated DEGs (Figures 1B,D). A heatmap revealed a distinct expression pattern for these 14 genes, effectively segregating the EP and HC groups (Figure 1C). GO and KEGG enrichment analysis of these genes highlighted their involvement in epigenetic regulation of gene expression, cellular energy metabolism and inflammation (Figures 1E,F).

### 3.2 Identification of TA-related DEGs for EP patients

**FIGURE 2:**
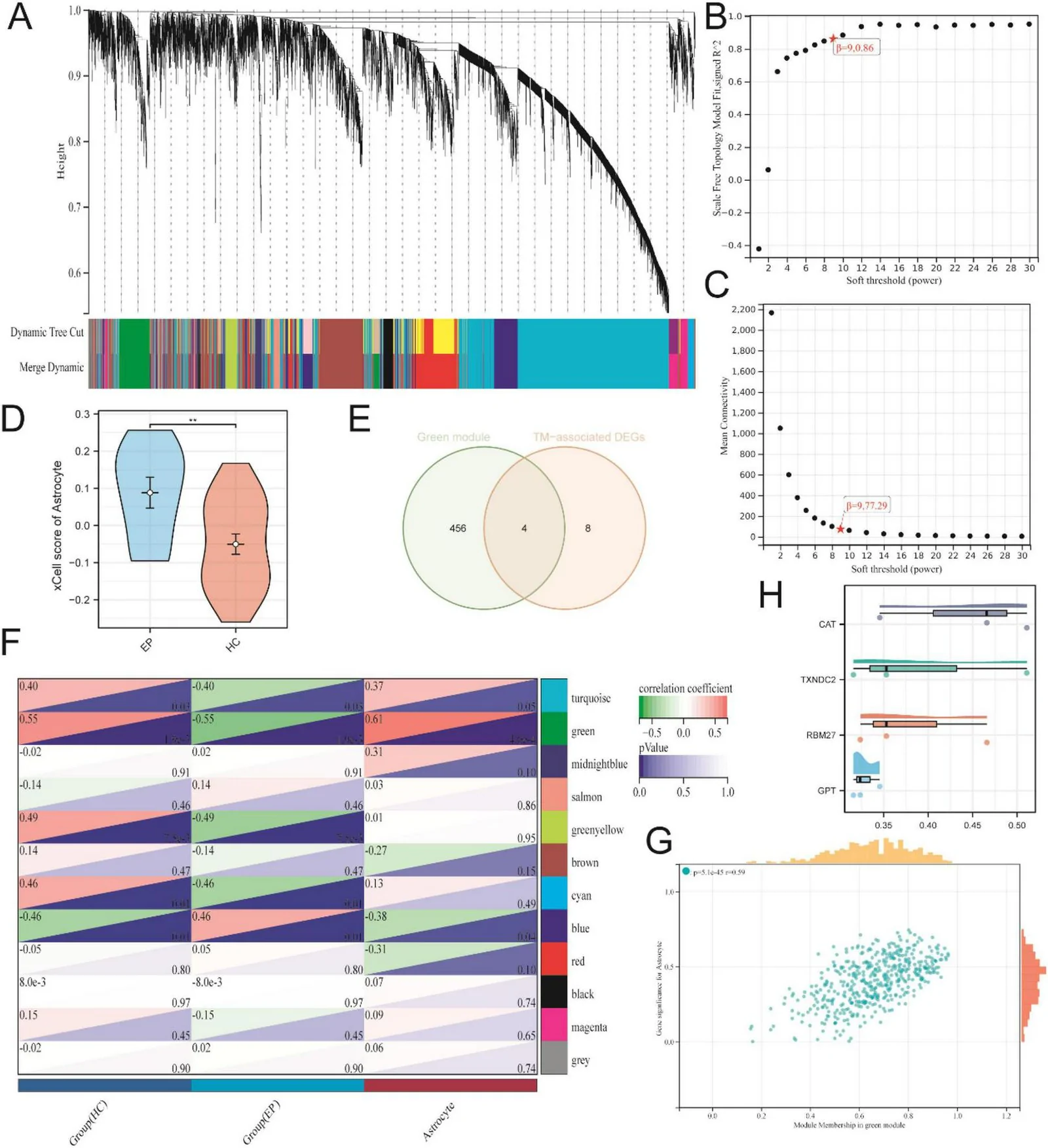
Identification of the TA-associated signature in EP. **(A)** WGCNA gene dendrogram and module assignment. **(B)** Scale-free topology fit index and **(C)** mean connectivity across soft-thresholding powers. **(D)** xCell violin plot showing greater astrocyte enrichment in EP. **(E)** Venn diagram showing the intersection that yielded four TA-associated DEGs. **(F)** Module–trait relationship heatmap, highlighting the green module as most strongly correlated with the astrocyte score and EP. **(G)** Correlation between module membership and gene significance in the green module. **(H)** Gene importance analysis of the four TA-associated DEGs. Results with *p* < 0.05 or FDR < 0.05 were considered significant.

xCell analysis of integrated GSE28674, GSE57585, and GSE256068 profile revealed a significant enrichment of astrocytes in EP samples compared to HCs (Figure 2D). WGCNA was then performed to find genes correlated with this astrocyte score. A soft threshold of β = 9 was selected (Figures 2A–C). The green module showed the strongest correlation with the astrocyte score (Figures 2F,G). Intersecting the 14 TM-associated DEGs with the 460 genes from the green module yielded 4 core TA-associated shared DEGs, including RBM27, GPT, CAT, and TXNDC2 (Figure 2H).

### 3.3 TA-associated molecular subgroup identification for EP patients

**FIGURE 3:**
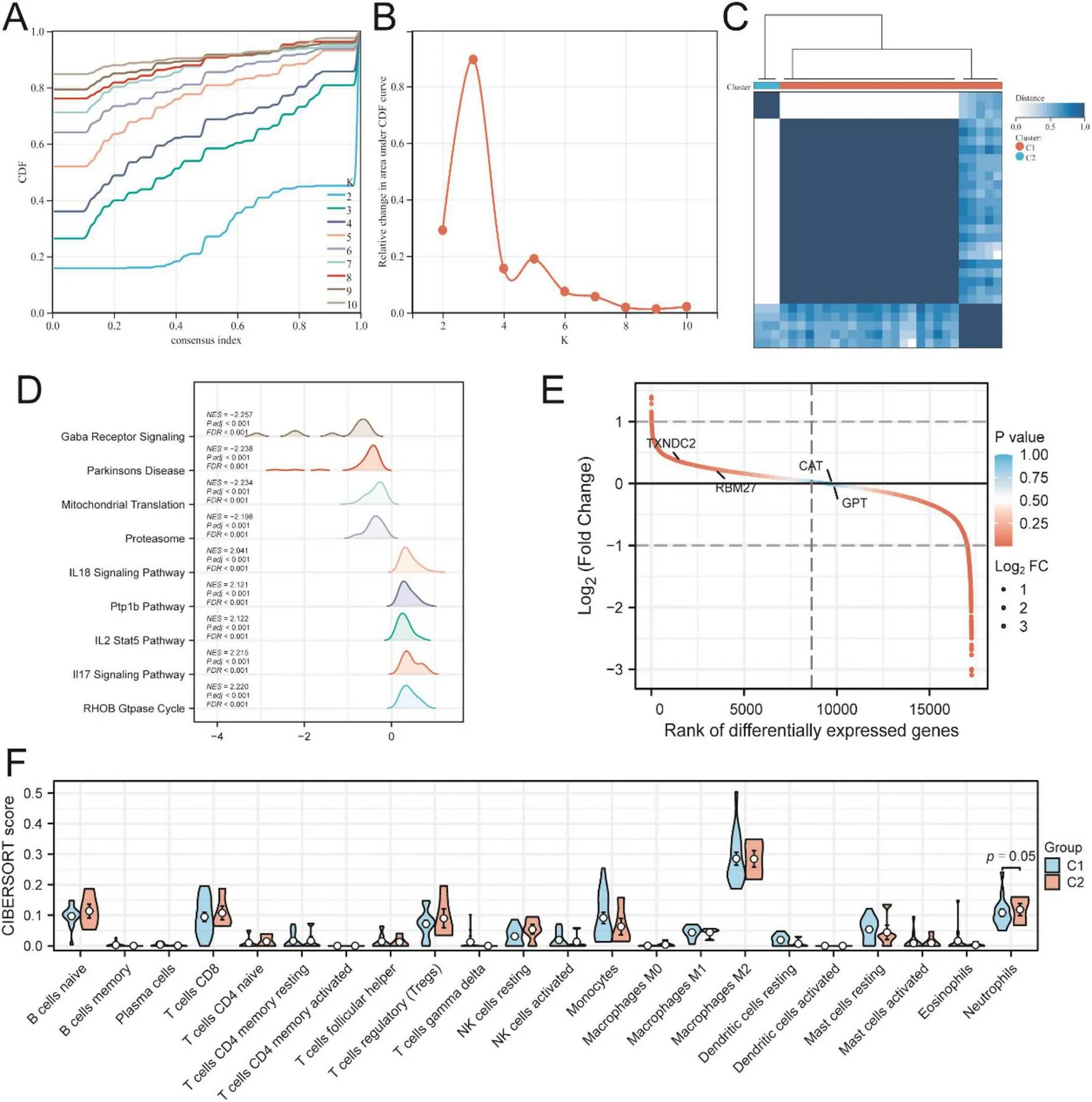
TA-associated signature-based molecular subtyping of patients with EP. **(A)** Consensus cumulative distribution function curves. **(B)** Relative change in the area under the CDF curve across cluster numbers. **(C)** Consensus matrix heatmap for *k* = 2, showing clear C1 and C2 clusters. **(D)** GSEA enrichment analysis comparing C1 and C2. **(E)** Ranked fold-change plot of differentially expressed genes, with the four TA-associated DEGs labeled. **(F)** CIBERSORT immune-infiltration comparison between C1 and C2. Results with *p* < 0.05 or FDR < 0.05 were considered significant.

Consensus clustering based on these 4 TA-associated shared DEGs in the GSE163296 cohort robustly stratified EP patients into two molecular subtypes, designated C1 and C2 (Figures 3A–C). The ranked fold-change plot showed higher expression of RBM27 and TXNDC2 in the C1 subtype (Figure 3E). GSEA revealed that the C1 subtype was enriched in pathways related to inflammation and synaptic signaling, whereas the C2 subtype was associated with metabolic homeostasis (Figure 3D). CIBERSORT analysis indicated a higher infiltration of activated neutrophils in the C2 subtype (Figure 3F).

### 3.4 TA-associated diagnostic model construction for EP patients

**FIGURE 4:**
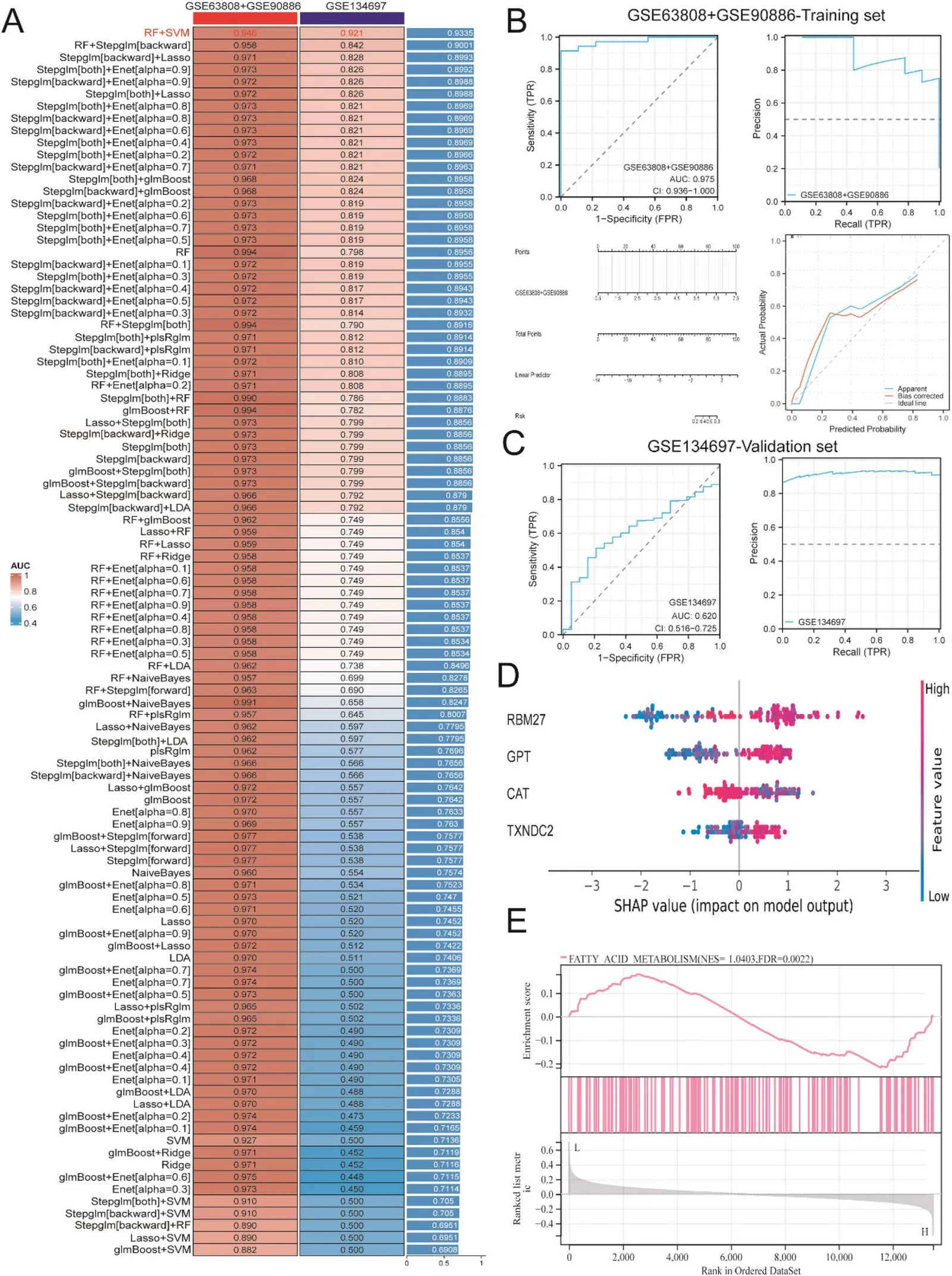
TA-associated diagnostic model construction and hub gene identification in EP. **(A)** Heatmap of AUC-index for 132 model combinations. **(B)** ROC and PR curves for the RF+SVM model with nomogram and calibration curve in the training set. **(C)** ROC and PR curves for the RF+SVM model in the validation set. **(D)** SHAP summary plot highlighting RBM27. **(E)** Single-gene GSEA plot for RBM27 in training set. Results with *p* < 0.05 or FDR < 0.05 were considered significant.

Using the 4 TA-associated DEGs, we trained 132 machine learning models on integrated GSE63808 and GSE90886, and GSE134697 (Figure 4A). The RF combined with SVM model exhibited the best performance, with a mean AUC-index of 0.9335 (Figure 4A). ROC and PR analysis confirmed robust diagnostic power in the integrated GSE63808 and GSE90886 (AUC = 0.975), which was successfully validated in the independent GSE134697, cohort (AUC = 0.620) (Figures 4B,C). A nomogram built from the model demonstrated good calibration in the integrated GSE63808 and GSE90886 (Figure 4B). SHAP analysis identified RBM27 as the most influential gene in the model (Figure 4D). Single-gene GSEA revealed that RBM27 is primarily involved in the positive regulation of fatty acid metabolism (Figure 4E).

### 3.5 Astrocyte molecular patterns at the single-cell level in patients with EP

**FIGURE 5:**
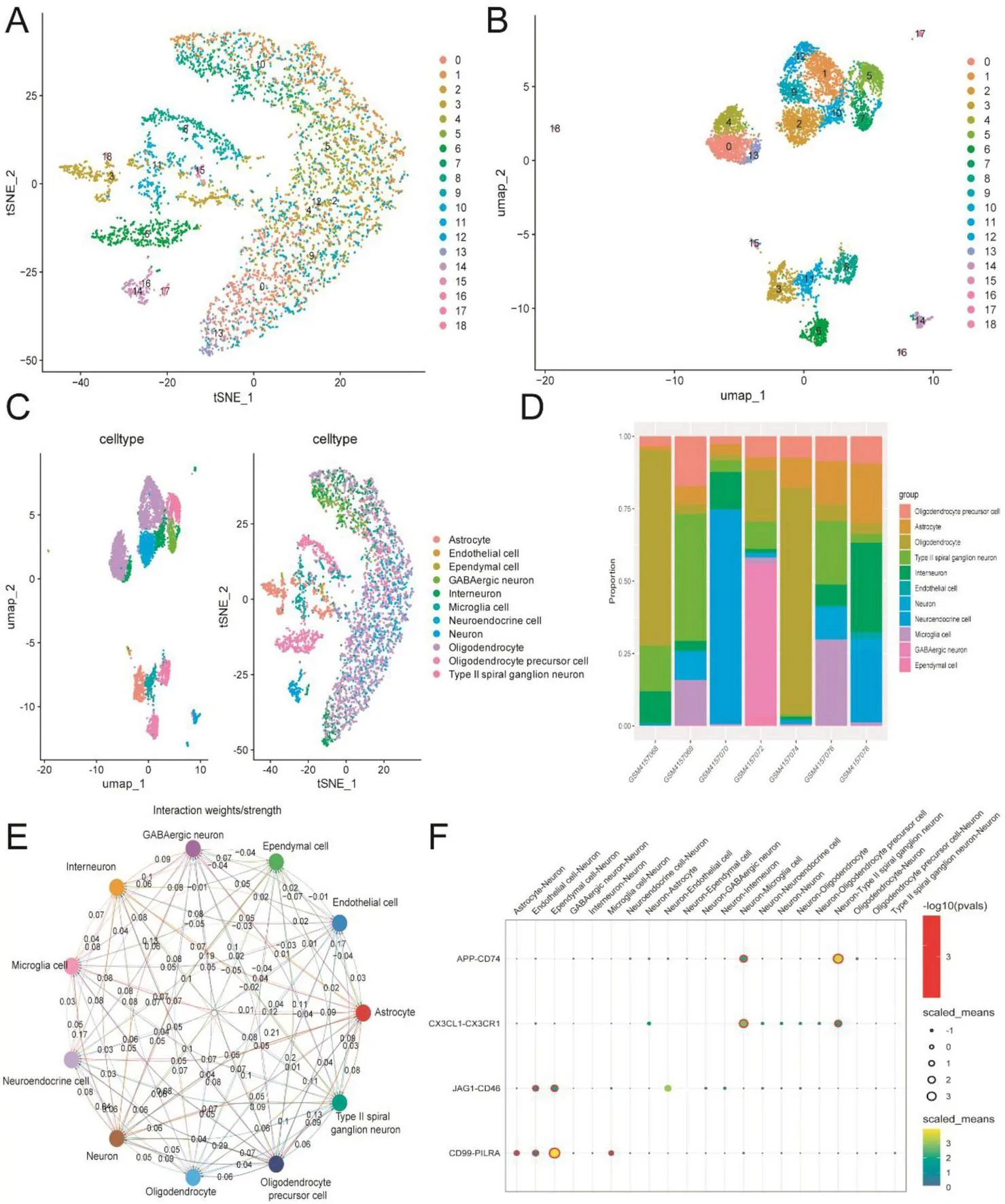
Single-cell landscape of astrocytes in the hippocampus of patients with EP. **(A)** t-SNE and **(B)** UMAP plots of 18 cell clusters. **(C)** Cell-type annotations on UMAP and t-SNE plots. **(D)** Cell-type proportions by sample. **(E)** CellChat network plot showing intercellular communication. **(F)** Dot plot of selected ligand–receptor interactions across cell-type pairs. Results with *p* < 0.05 or FDR < 0.05 were considered significant.

Analysis of scRNA-seq data (GSE190452) resolved 18 cell clusters, which were annotated into 11 major cell types (Figures 5A–D). CellChat analysis revealed strong intercellular communication involving endothelial cells, which acted as a critical hub for signaling with neurons and glial cells (Figures 5E,F).

### 3.6 TA-associated molecular patterns in astrocytes at the single-cell level in patients with EP

**FIGURE 6:**
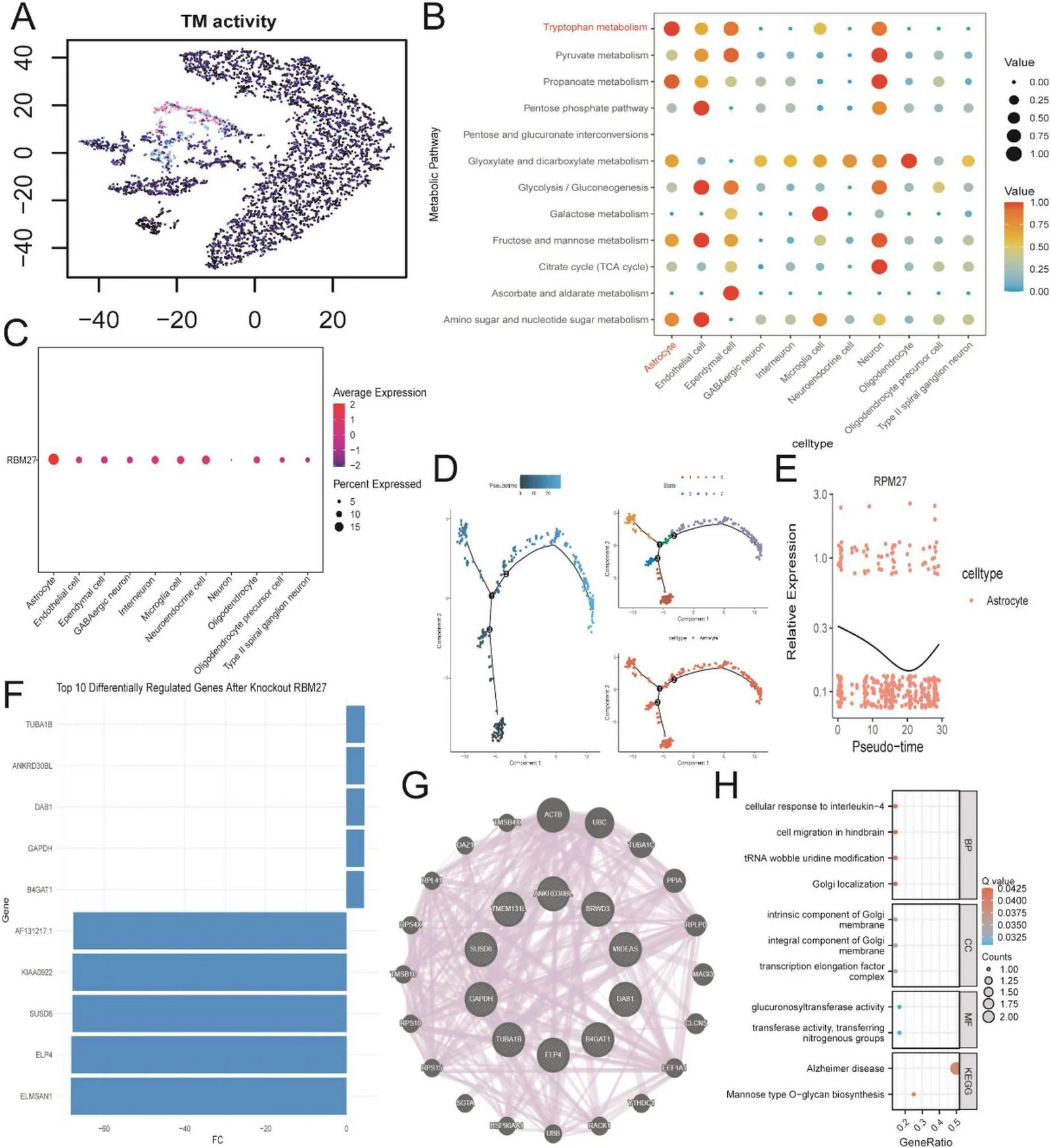
TA-associated molecular patterns in astrocytes of patients with EP. **(A)** AUCell score for the TM pathway across cell types, showing the highest activity in astrocytes. **(B)** Dot plot of metabolic pathway activity highlighting TM activation in astrocytes. **(C)** Dot plot showing RBM27 expression enriched in astrocytes. **(D,E)** Pseudotime trajectories of astrocytes, showing seven branches and RBM27 expression along pseudotime. **(F)** Top 10 differentially regulated genes after in silico RBM27 knockout. **(G)** GENEMANIA interaction network of genes associated with the RBM27-knockout signature. **(H)** GO and KEGG enrichment of perturbed pathways following in silico RBM27 knockout. Results with *p* < 0.05 or FDR < 0.05 were considered significant.

AUCell analysis showed that TM activities were significantly higher in astrocytes (Figure 6A). Metabolic profiling showed distinct heterogeneity among cell clusters, with astrocytes displaying a unique TM signature (Figure 6B). Feature plots confirmed that the hub gene RBM27 was predominantly expressed in astrocytes (Figure 6C). An *in silico* knockout of RBM27 specifically within astrocytes perturbed a network of genes enriched in pathways related to neurodegeneration, inflammatory response, and epigenetic modification (Figures 6F–H). Monocle2 pseudotime analysis of astrocytes revealed seven different differentiation trajectories, with RBM27 expression gradually decreasing along the main trajectory toward a more diseased state, indicating that these results suggest a potential negative regulatory role for RBM27 in astrocyte differentiation (Figures 6D,E).

### 3.7 Therapeutic candidate identification and clinical validation targeting TA-associated molecular signature for EP patients

**FIGURE 7:**
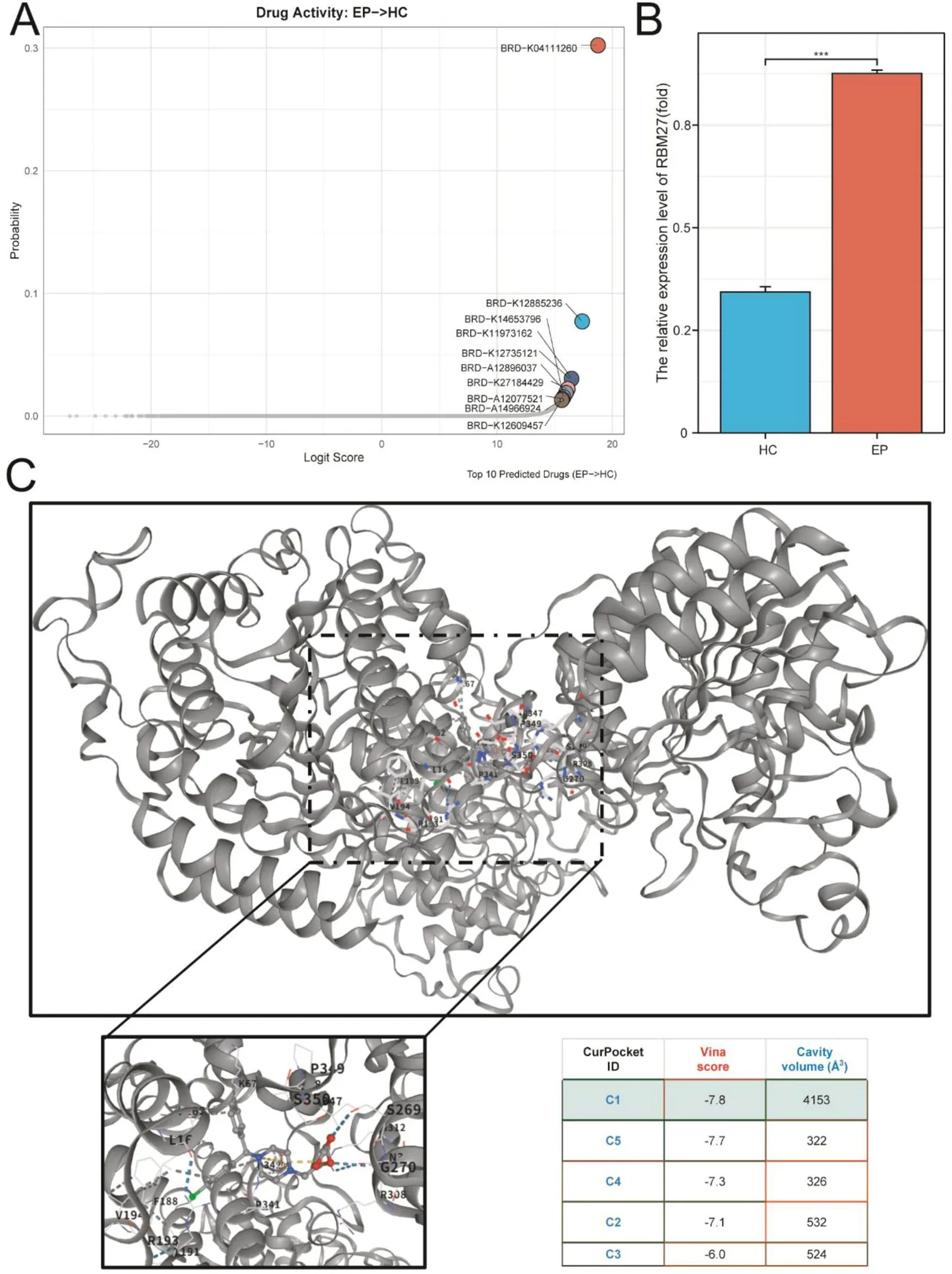
Drug prediction and clinical validation targeting RBM27 in EP. **(A)** DrugReflector ranking of top compounds, showing BRD-K04111260 as the top candidate. **(B)** q-RT-PCR validation of RBM27 mRNA expression in EP compared to control human hippocampal tissue. **(C)** Molecular docking model of BRD-K04111260 binding to RBM27. Results with *p* < 0.05 or FDR < 0.05 were considered significant.

Drug repositioning using DrugReflector identified BRD-K04111260 (Raclopride) as the top-ranked compound with DTS = 0.95 predicted to reverse the EP-associated gene expression signature (Figure 7A). Molecular docking analysis between BRD-K04111260 and the protein structure of RBM27 predicted a strong binding affinity (binding energy = –7.8 kcal/mol) (Figure 7C). Finally, qPCR validation in clinical hippocampal samples confirmed that RBM27 mRNA levels were significantly elevated in EP patients compared to controls, substantiating our bioinformatics findings (Figure 7B).

## 4 Discussion and conclusion

In this study, we conducted a systematic multi-omics investigation to elucidate the role of TA-associated patterns during epileptogenesis. By integrating bulk transcriptomics, single-cell analysis, and an ensemble machine-learning framework of 132 models, we identified a TA-associated signature and constructed a robust diagnostic model. Our central finding is the identification of RBM27 as the pivotal hub gene connecting TM dysregulation to astrocyte-mediated neuroinflammation and metabolic disturbance in EP.

RBM27 (RNA-binding motif protein 27) is a member of the RNA-binding protein family, involved in post-transcriptional regulation of gene expression, including RNA splicing, stability, and localization (Huang et al., 2023). Its molecular functions are critical for various cellular processes, including cell cycle control, differentiation, and stress response (Chowdhury et al., 2024a). In the context of neurological and psychiatric diseases, RBM27 has been implicated in the regulation of synaptic plasticity and neuronal survival (Deng et al., 2023). For instance, RBM27 has been shown to interact with Fragile X messenger ribonucleoprotein 1 (FMRP) to regulate dendritic mRNA translation (Chowdhury et al., 2024b). More recently, studies have linked RBM27 mutations and dysregulation to autism spectrum disorder and schizophrenia, suggesting a broader role in neurodevelopment (Chowdhury et al., 2024a). Within EP, direct evidence is limited, but our findings provide compelling computational and experimental data positioning RBM27 at the interface of metabolism and neuroinflammation. Our single-gene GSEA analysis revealed a positive correlation between RBM27 and fatty acid metabolism, which is noteworthy given that altered brain energy metabolism is a well-known feature of EP. The *in silico* knockout of RBM27 in astrocytes perturbed pathways related to neuropsychiatric disease and inflammation, strongly suggesting that RBM27 acts as a master regulator of astrocyte reactivity in the epileptic brain. Our pseudotime analysis, revealing decreased RBM27 expression along astrocyte differentiation, further hints at a potential role in astrocyte maturation or acquisition of a reactive phenotype.

This study delineates a novel TA-associated molecular axis in EP pathogenesis through an integrative multi-omics framework. By combining Limma, WGCNA analysis, consensus clustering and an ensemble machine-learning pipeline of 132 models, we identified a four-gene TA-associated signature for diagnosis and molecular stratification for EP patients, with RBM27 emerging as the central hub. Single-cell profiling confirmed that RBM27 is predominantly expressed in astrocytes, where its *in silico* knockout perturbed neuropsychiatric, inflammatory, and epigenetic pathways, underscoring its role as a master regulator of astrocyte dysfunction. Drug repositioning identified BRD-K04111260 as a promising compound targeting RBM27, and clinical q-RT-PCR validation in hippocampal specimens confirmed elevated RBM27 expression in EP patients. Collectively, these findings establish RBM27 as a critical link between TM dysregulation and astrocyte-mediated neuroinflammation in EP, providing a robust diagnostic biomarker and a novel therapeutic target warranting further preclinical investigation. Nevertheless, our study has several limitations. Although batch effect correction was applied, the reliance on public transcriptomic cohorts introduces inherent technical and biological noise that may not be eliminated. To strengthen the clinical applicability of our diagnostic model, large-scale, multicenter prospective trials are warranted to rigorously validate the diagnostic and molecular stratification algorithms developed herein. In parallel, while our single-cell analysis affords high-resolution granularity, it is derived from a modest cohort of 4 EP patients, which may not fully encompass the diverse astrocytic states across the EP continuum. Integrating state-of-the-art AI with higher-resolution spatial and single-cell technologies, alongside dynamic *in vivo* tracing of TM dynamics within astrocytes, would substantially advance our understanding of EP pathogenesis. Mechanistically, the precise downstream signaling cascades through which astrocytic RBM27 impacts neuronal integrity via TM regulation remain incompletely resolved. Although in a different disease context, target-directed gene delivery has demonstrated therapeutic efficacy in a large-animal model, as exemplified by cardiac BIN1 gene therapy for chronic non-ischemic heart failure (Li et al., 2024). Moving forward, conditional knockout or astrocyte-specific RBM27 overexpression models in EP mice will be essential for establishing causality and corroborating the *in silico* perturbation findings. In addition, the clinical validation sample size was relatively small. Future studies should examine RBM27 function in large, multicenter cohorts of hippocampal tissue from patients with EP. Additionally, high-throughput screening for astrocyte-selective RBM27 modulators may offer more refined therapeutic avenues than conventional pan-inhibitors such as statins. Regarding the computationally nominated candidate BRD-K04111260, comprehensive preclinical evaluations, including pharmacokinetic profiling, blood-brain barrier permeability testing, and verification of its impact on the TA-related molecular pattern and RBM27 activity *in vivo*, are urgently required prior to further clinical translation.

## Data Availability

The datasets presented in this study can be found in online repositories. The names of the repository/repositories and accession number(s) can be found in the article/supplementary material.
